# Geochemical Characteristics and Origins of the Crude Oil of Triassic Yanchang Formation in Southwestern Yishan Slope, Ordos Basin

**DOI:** 10.1155/2017/6953864

**Published:** 2017-07-02

**Authors:** Xiaoli Zhang, Jinxian He, Yande Zhao, Hongchen Wu, Zeqiang Ren

**Affiliations:** ^1^School of Resources and Geoscience, China University of Mining and Technology, Xuzhou 221116, China; ^2^Key Laboratory of Coalbed Methane Resource and Reservoir Formation Process, Ministry of Education, China University of Mining and Technology, Xuzhou 221116, China; ^3^Research Institute of Petroleum Exploration and Development, PetroChina Changqing Oilfield Company, Xi'an, Shanxi 710018, China

## Abstract

Biomarker compounds that derived from early living organisms play an important role in oil and gas geochemistry and exploration since they can record the diagenetic evolution of the parent materials of crude oil and reflect the organic geochemical characteristics of crude oil and source rocks. To offer scientific basis for oil exploration and exploitation for study area, gas chromatography-mass spectrometry method is applied to study the biomarker compounds of crude oil in Southwestern Yishan Slope of Ordos Basin, through qualitatively and quantitatively analyzing separated materials. The crude oil of Yanchang Formation and the source rocks of Yan'an and Yanchang Formation were collected in order to systematically analyze the characteristics of the biomarker compounds in saturated hydrocarbon fractions and clarify the organic geochemical characteristics of crude oil. The distribution and composition of various types of hydrocarbon biomarker compounds in crude oil suggest that the parent materials of crude oil are composed of hydrobiont and terrigenous plants, and the crude oil is mature oil which is formed in the weak reducing fresh water environment. Oil source correlation results show that the crude oil of Yanchang Formation in Yishan Slope is sourced from the source rocks of Chang 7 subformation.

## 1. Introduction

Biomarker compounds that occur in sedimentary organic matter are derived from early living organisms and are stable in the evolution of organic matters. They record the complex organic compounds of original biological parent materials. The maturity of the chromatographic-mass spectrometry technology has ensured the important position of biomarker compounds in oil and gas geochemistry and exploration. Biomarker compounds with characteristic stereochemical structures and rich information have both inheritance and variability in the evolution process of organisms, sedimentary organic matter, oil, and gas. Although the biomarker compounds in crude oil are low in abundance, they are diversified in types and extensive in distribution. Some biomarker compounds with very good specificity and strong mark function are of important significance in judging the source, depositional environment, and maturity of organic matter and studying oil source correlation and hydrocarbon migration [1]. Gas chromatography-mass spectrometry (GC-MS) analysis techniques is the most important method to study the biomarker compounds.

**Figure 1:**
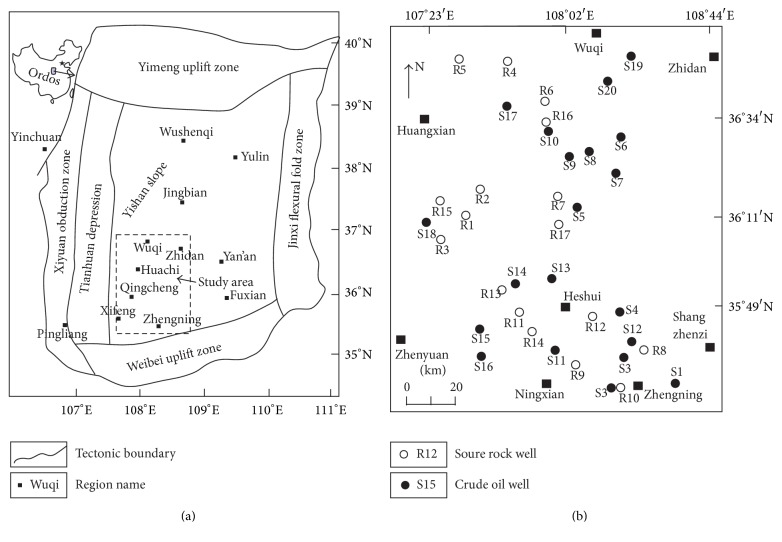
Tectonic units of the Ordos Basin and locations of crude oil and source rock samples.

The Ordos Basin as the second largest petroliferous sedimentary basin in China can be divided into six secondary structural units. As the largest secondary structural unit in the Ordos Basin (Figure 1), Yishan Slope is the focus for oil and gas exploration and development in the basin. Triassic Yanchang Formation is the major exploration target in the basin, which is divided into ten oil groups (Chang 1 to Chang 10 from top to bottom) by oil and gas exploration department. Important breakthroughs have been made in the exploration and development of the crude oil in the Ordos Basin and good achievements have been gained in the geochemical study of Mesozoic crude oil [2–7]. However, because of the scattered geochemical study and uneven distribution of the oil groups of source rock samples [8–11], a systematic study on the geochemistry with abundant samples of crude oil and source rock distributed reasonably in Yanchang Formations is necessary.

In this study, the crude oil samples of Chang 1 to Chang 10 and the source rock samples of Jurassic Yan'an Formation and Yanchang Formation Chang 4 + 5, Chang 6, 7, 8, and Chang 9 were systematically collected to study the geochemistry of crude oil and discuss parent material characteristics, formation environment, and maturity of crude oil, which can provide geochemical evidence for the study on hydrocarbon accumulation.

## 2. Sampling and Experimentation

### 2.1. Sampling

Crude oil samples of 20 wells and black mudstone samples of 17 wells were systematically collected from the Southwestern Yishan Slope, Ordos Basin. The well location is shown in Figure 1, and the data of group components of crude oil and source rock samples was shown in Table 1.

### 2.2. Experimentation

#### 2.2.1. Soxhlet Extraction

*Instrument*. Soxhlet extractor is composed of three parts, including flask, extractor, and reflux condenser.

*Experimental Conditions*. The mudstone samples were ground to 100 mesh (0.150 mm) before extraction. 100 g of crushed mudstone, which weighed into a filter cartridge, was extracted using a Soxhlet apparatus. 300 mL chloroform and 2 pieces of red copper slices were added to the flask that was connected to extractor. The flask was heated in water-bath at a temperature of 78–82°C for extracting. Starting from the reflux of chloroform, extraction should last for 72 hours. After extraction, the flask was moved to water-bath kettle to concentrate the leaching liquor. Then, the funnel with degreasing cotton was used to filter. Eventually, the solvent was evaporated in oven at a constant temperature of 40°C until a constant weight.

#### 2.2.2. Group Component Separation

*Instrument*. Glass chromatographic column.

*Experimental Conditions*. 15 mL n-hexane was added to 30 mg extract to dissolve for 12 hours. Then asphaltene was removed through filtration and the remaining solution without asphaltene was naturally volatilized to 2 mL. Transfer the 2 mL sample into a glass chromatographic column with 3 g silica gel (60 meshes) and 2 g alumina (100 meshes) in it. The saturated hydrocarbon fraction was collected after leaching with 20 mL n-hexane for four times; the aromatic fraction was collected after leaching with dichloromethane-n-hexane (2 : 1 by volume) and the nonhydrocarbon fractions were collected after leaching with anhydrous ethanol solution.

#### 2.2.3. Chromatography-Mass Spectrometry (GC-MS) Test

*Instrument*. Agilent GC6890N/MS5973N.

*Chromatograph Experimental Conditions*. The injection volume is 0.2 *μ*L. Split ratio is 30 : 1 and precolumn pressure is 11 standard atmospheric pressures. The chromatographic column is HP-5MS column (30 m × 0.32 mm) with the coating thickness of immobile phase is 0.25 *μ*m and carrier gas is helium. The experimental temperature rises from 80°C to 300°C at a rate of 4°C/min and then keeps for 30 min.

*Mass Spectrometry Experimental Conditions*. EI ionization mode was adopted, with electron multiplication voltage of 900 V, scanning range of 18–690 AMV, ionization voltage of 70 eV, ion source temperature of 250°C, and GC/MS interface temperature of 300°C.

## 3. Results and Discussion

The biomarker compounds in the saturated hydrocarbon fraction of crude oil are high in abundance and have obvious characteristics. They are reliable indexes for the evaluation of crude oil, since they can stably reflect the depositional environment, the composition of raw material, the preservation condition, and the thermal evolution information of the parent material of crude oil. N-alkanes, isoprenoid alkanes, Steranes, and Terpanes are mainly used as saturated hydrocarbon biomarker compounds. Based on these parameters, the geochemical characteristics of crude oil can be reflected more comprehensively. Therefore, this paper focuses on the characteristics of n-alkanes, isoprene alkanes, Steranes, and Terpanes.

### 3.1. Characteristics of n-Alkanes and Isoprenoid Alkanes

The chromatographic features of n-alkane contain rich information about sedimentary environment, source composition, preservation condition, and thermal evolution [12–16]. N-alkanes are saturated straight-chain alkanes. The n-alkanes with different sources have different ranges of carbon numbers: the prepeak n-alkanes with C_15_–C_21_ are mainly sourced from lower organism, while the postpeak n-alkanes with C_23_–C_35_ are mainly sourced from higher organism. At diagenetic stage, deacidification exceeds reduction in oxidizing environment and the carbon number of hydrocarbons is one less than that of biological organic matter in the process of linear acids and alcohols converting to hydrocarbons and odd carbon advantage occurs in hydrocarbons. On the contrary, reduction takes dominance; even carbon advantage will appear. With the increase of maturity, carbon dominance gradually disappears and the ratio between prepeak and postpeak (OEP) approaches 1.

The acyclic isoprene alkanes are the paraffins composed of isoprenoid structural units. The compounds with low carbon number (lower than C_20_) are phytane series compounds, which are the strong reduction products of the pigment in higher plants or photosynthetic bacteria. The phytate or phytane in the pigment material begins to form pristane under oxidation with the weakening of the reduction conditions. Accordingly, the ratio of pristane to phytane (Pr/Ph) can be used to determine the depositional environment. It is generally acknowledged that Pr/Ph < 0.5 signifies strong reducing sedimentary environment [17] and Pr/Ph = 0.5~1.0 and Pr/Ph = 1.0~2.0 suggest reducing and weak reducing-weak oxidizing environment, respectively, while Pr/Ph > 2.0 indicates oxidizing environment [17, 18].

**Figure 2:**
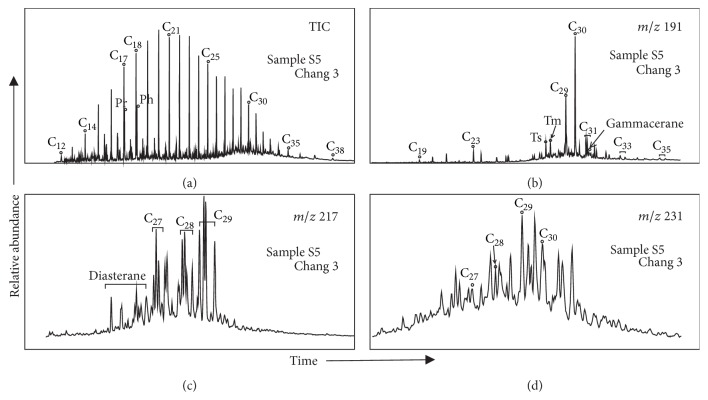
Representative chromatograms of n-alkanes (a), Terpanes (b), Steranes (c), and diasterane (d) of the crude oil of Yangchang Formation in Southwestern Yishan Slope.

**Figure 3:**
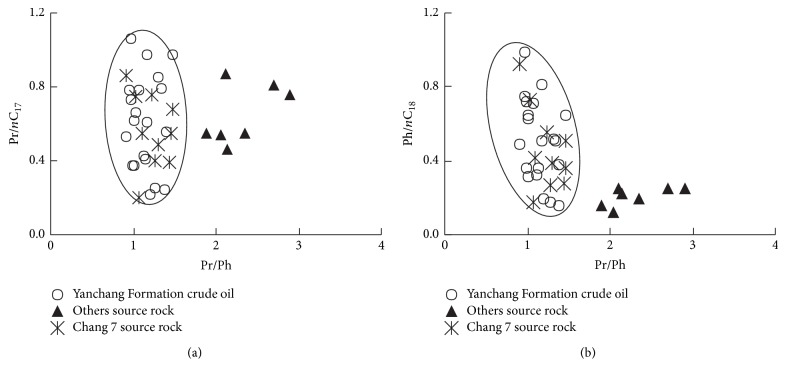
Cross plots of Pr/*n*C_17_ versus Pr/Ph ratios (a) and Ph/*n*C_18_ versus Pr/Ph ratios (b) of samples in Southwestern Yishan Slope.

The crude oil samples have the same distribution characteristics of n-alkane in this study (Figure 2). The carbon number distribution is C_11_~C_38_, the parity advantage is not obvious, the maximum carbon number is C_17_~C_21_, and ∑C_21_^−^/∑C_21_^+^ ranges from 0.77 to 2.47 (Table 2), indicating that the crude oil is composed by hydrobiont and terrigenous plants and the aquatic bacteria play an important role. In addition, the values of Pr/*n*C_17_ and Ph/*n*C_18_ distribute in a narrow range, suggesting crude oil comes from a similar biological source [2, 12, 19] (Figure 3).

There is no parity advantage in the n-alkanes of crude oil and the value of OEP ranges from 1.01 to 1.09, closed to 1.0, indicating that the crude oil is mature. The Pr/Ph of the crude oil of Yanchang Formation and the source rocks of Chang 7 in study area ranges from 0.91 to 1.47 with relative concentrated distribution. Pristanes (Pr) and Phytane are not dominated, reflecting weak reducing-weak oxidizing sedimentary environment. The Pr/Ph of other source rocks ranges from 1.89 to 2.90, which indicates oxidizing sedimentary environment.

**Figure 4:**
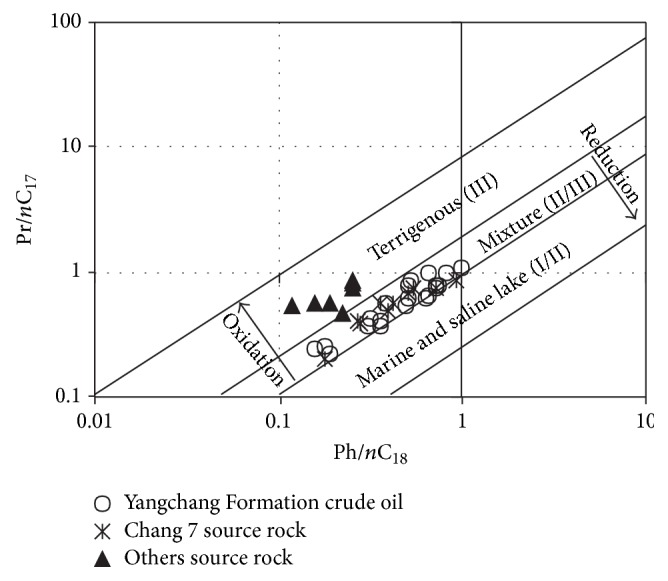
Cross plots of Pr/*n*C_17_ versus Ph/*n*C_18_ ratios of crude oil and source rocks in Southwestern Yishan Slope.

The distribution of Pr/*n*C_17_ and Ph/*n*C_18_ of crude oil is usually used to study the types of parent materials, formation environment, and maturity [12, 14, 19–21]. As shown is Figure 4 the values of Pr/*n*C_17_ and Ph/*n*C_18_ distribute in a narrow range, showing the crude oil comes from similar parent materials and similar formation environments. It is also shown in Figure 4 that the data points of the crude oil of Yanchang Formation and the data points of the source rocks of Chang 7 fall in the same range of weak reducing-weak oxidizing environment, while the other source rocks are in a oxidizing terrestrial environment, indicating the crude oil is mainly derived from the source rocks of Chang 7, and the contribution of other source rocks (the general term for the source rocks of Yan'an Formation, Chang 4 + 5, Chang 6, Chang 8, and Chang 9, below is the same) is not significant.

### 3.2. Composition Characteristics of Steranes

The distribution of the Steranes in crude oil can reflect the sources of the parent materials of crude oil [22–25]. The values of ∑⁡regular  Steranes/∑⁡Hopanes are usually used to represent the inputs of eucaryote (mainly alga and advanced plant) and procaryote (bacterium) in the parent materials of crude oil. The high ∑regular  Steranes/∑Hopanes (≥1) value represents the marine organic characteristics of alga [23], while the low ∑regular  Steranes/∑Hopanes value is the characteristics of terrigenous organic matter or the organic matter modified by microorganisms [26]. The content of diasteranes increases with the maturity of crude oil. Therefore, the value of ∑diasteranes/∑regular Steranes can be used to evaluate the maturity of crude oil [27].

**Figure 5:**
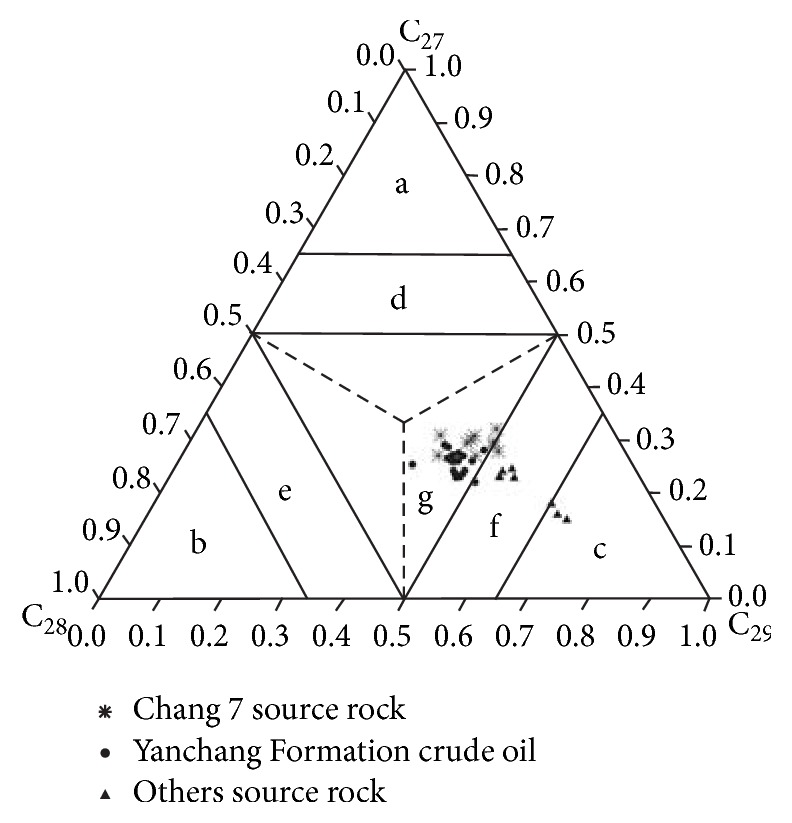
Distribution triangular chart of different carbon number regular Steranes of crude oil and source rock samples in Southwestern of Yishan Slope. (a-plankton; b-alga; c-terrestrial plant; d-dominated by phytoplankton; e-dominated by alga; f-dominated by terrestrial plant; g-mixed source).

The regular Steranes in the crude oil of Yanchang Formation have similar distribution to that of the source rock of Chang 7 and different from that of other source rocks in Southwestern Yishan Slope. In the crude oil of Yanchang Formation, the C_29_ Steranes in regular Steranes have the highest content (38.6~50%), but the contents of C_27_ Steranes (23~31%) and C_28_ Steranes (23~36%) are similar (Table 3). The three peaks showed a reversed “L” shape, indicating the parent materials of crude oil are from mixed source (Figure 5) and the contribution of terrestrial higher plant is great. In the source rocks of Chang 7, the C_29_ Steranes in regular Steranes have the highest content (40.4~51.4%) and C_28_ Steranes (26.9~30.8%) and C_28_ Steranes (19~31%) have similar content, while, in other source rocks, the content of C_29_ Steranes in regular Steranes has the highest content (52~69%) and C_27_ Steranes (15~23%) and C_28_ Steranes (16~23%) have similar content (Table 3). It can be concluded that the crude oil of the Yanchang Formation and the source rock of Chang 7 are comparable, while significantly different from other source rocks.

**Figure 6:**
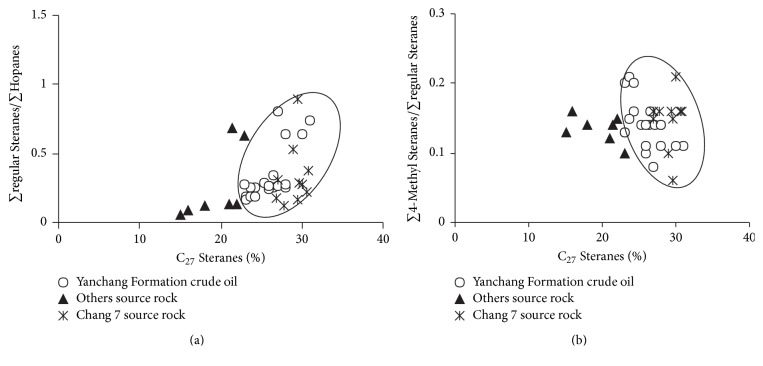
Cross plots of ∑regular  Steranes/∑Hopanes versus C_27_ Steranes ratios (a) and ∑4-Methyl Steranes/∑regular Steranes versus C_27_ Steranes ratios (b) of the crude oil and source rock samples in Southwestern of Yishan Slope.

The ∑regular Steranes/∑Hopanes of crude oil in the Yanchang Formation are low that range from 0.16 to 0.80, indicating that terrigenous higher plants make an important contribution to the parent materials and microbe degradation may occur weakly at the same time. 4-Methyl Steranes, which usually come from Dinoflagellata [28] and bacterium [24], are relatively abundant in the Yanchang Formation in Southwestern Yishan Slope. The value of ∑4-Methyl Steranes/∑regular Steranes ranges from 0.08 to 0.21, indicating that Dinoflagellata and bacterium also contribute to the formation of crude oil in study area. The above-mentioned ratios are in concentrated distribution (Figure 6), suggesting similar sources of parent materials and the parent materials are mixed typed.

The value of C_29_ diasteranes 20S/(20S + 20R) in the Yanchang Formation crude oil is between 0.45 and 0.77 and ∑diasteranes/∑regular Steranes ranges from 0.07~0.75, reflecting the crude oil is mature.

**Figure 7:**
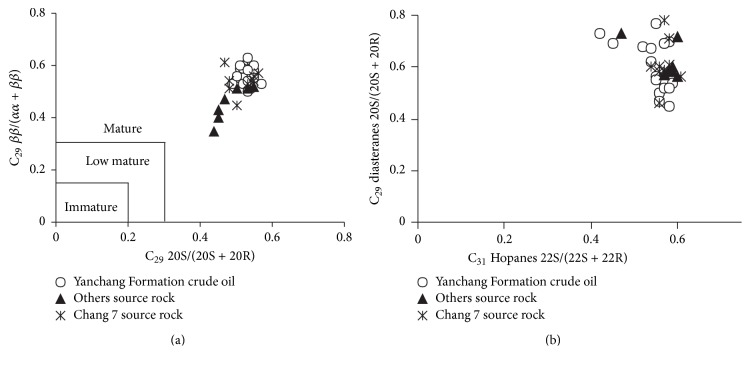
Cross plots of C_29_ Steranes *ββ*/(*αα* + *ββ*) versus C_29_ Steranes 20S/(20S + 20R) (a) and C_29_ diasteranes 20S/(20S + 20R) versus C_31_ Hopanes 22S/(22S + 22R) (b) of the crude oil and source rock samples in Southwestern of Yishan Slope.

Previous study [29] proposed that the values of C_29_ Steranes 20S/(20S + 20R) and *ββ*/(*αα* + *ββ*) in mature crude oil are bigger than 0.4. The distribution ranges of C_29_ Steranes 20S/(20S + 20R) and *ββ*/(*αα* + *ββ*) in crude oil are from 0.49 to 0.57 and from 0.50 to 0.63, respectively. They distribute in a relative narrow range, as shown in Figure 7(a), indicating that the maturity of the crude oil of Yanchang Formation is similar and the crude oil is mature.

### 3.3. Characteristics of Terpanes Composition

Tricyclic Terpanes and tetracyclic Terpanes are abundant in crude oil, which can well reflect the nature of parent materials [30]. The (C_19_ + C_20_)/C_23_ and C_25_/C_26_ values of tricyclic Terpanes can be used to judge the parent materials of crude oil [21, 31]. In marine crude oil, the C_25_/C_26_ value of tricyclic Terpanes is bigger than 1, while in terrestrial crude oil, it is smaller than 1 [32]. The *β*-dancane value can reflect of the oxidability of the formation environment of crude oil. The relative amount of gammacerane is positively correlated to the sedimentary paleosalinity [23, 24, 33, 34], which is an important index to characterize water salinity. The value of C_31_ Hopanes 22S/(22S + 22R) can reflect the maturity of crude oil [21, 31, 34, 35].

**Figure 8:**
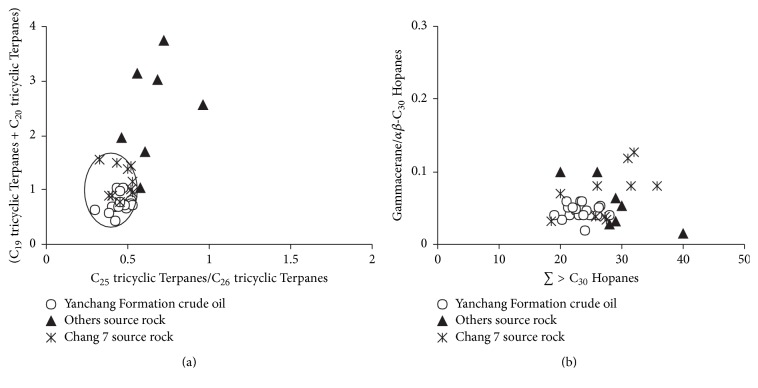
Cross plots of (C_19_ tricyclic Terpanes + C_20_ tricyclic Terpanes)/C_23_ tricyclic Terpanes versus C_25_ tricyclic Terpanes/C_26_ tricyclic Terpanes (a) and gammacerane/*αβ*-C_30_ Hopanes versus *∑* > C_30_ Hopanes ratios (b) of the study crude oil and source rock samples in Southwestern of Yishan Slope.

The (C_19_ + C_20_)/C_23_ and C_25_/C_26_ values of tricyclic Terpanes in the Yanchang Formation crude oil are concentrated and range from 0.44 to 1.05 and 0.30 to 0.53, respectively (Table 4, Figure 8(a)), manifesting the similar parent materials of crude oil of mixed type, in which terrestrial organic matter plays an important role. C_24_ tetracyclic Terpanes/(C_24_ tetracyclic Terpanes + C_26_ tricyclic Terpanes) range from 0.23 to 0.51, which also indicates that the parent materials of higher plant have great contribution on Yanchang Formation crude oil.

The low of *β*-dancane value in the crude oil suggests that the formation environment is weak reducing-weak oxidizing. The distribution ranges of gammacerane/*αβ*-C_30_ Hopanes and *∑* > C_30_ Hopanes relative amount are from 0.02 to 0.06 and from 19 to 28%, respectively, which are concentrated, as shown in Figure 8(b), indicating the crude oil of Yanchang Formation is formed in a similar low salinity environment.

The value of C_31_ Hopanes 22S/(22S + 22R) can reflect the maturity of crude oil [21, 31, 34, 35], which ranges from 0.52 to 0.61 (Table 4, Figure 7(b)) and reaches an equilibrium value of 0.57~0.64, indicating that the studied crude oil is mature. The same result can be got from the ratios of Ts/Tm, which are bigger than 0.5.

### 3.4. Oil Source Correlation Analysis

It can be concluded from the above-mentioned data that the crude oil of Yanchang Formation in Southwestern Yishan Slope is sourced from similar parent materials. In addition, the results of multiple tests and analyses suggest that these crude oils and the Chang 7 source rocks of the Yanchang Formation are very comparable as they have similar source materials, hydrocarbon generation environment, and maturity, while they are different from the other source rocks obviously in the important oil source correlation index (Pr/Ph) and the relative content of C_29_ Steranes.

**Figure 9:**
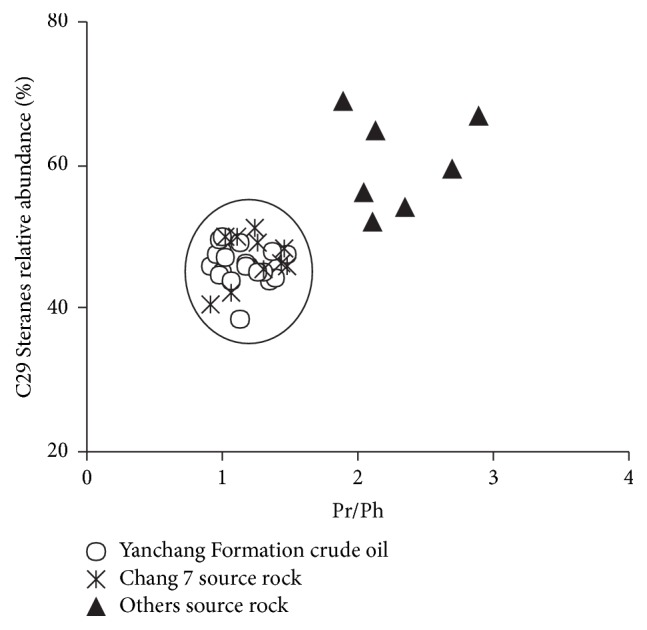
Cross plots of contents of C_29_ Steranes (%) versus Pr/Ph ratios of the crude oil and source rock samples in Southwestern of Yishan Slope.

The value of Pristanes/Phytanes (Pr/Ph) in n-alkanes and the relative content of C_27_, C_28_, and C_29_ in regular Steranes are important oil source correlation indexes, while the correlation diagram of Pr/*n*C_17_ versus Ph/*n*C_18_ can reflect the types of parent materials and formation environment directly and it also can be used as an index for oil source correlation. It can be found intuitively in Figures 4 and 9 that the crude oil of the Yanchang Formation and the source rocks of Chang 7 distribute in a relative narrow range, different from other source rocks, suggesting a genetic relationship in the studied area.

What is more, n-alkanes and isoprenoid hydrocarbon in the crude oil and Chang 7 source rocks are similarly distributed, whose carbon number distribution ranges broadly with one single peak; the terpenoid and steroid are similar obviously, the content of tetracycline is high, the homohopanes with high carbon number appears in pairs, the content of tricyclene is low, and the content of regular Steranes C_29_ is higher than C_27_ in steroid. It can be also found intuitively from the mass chromatogram that the distribution characteristics of the crude oil and the Chang 7 source rocks are similar, both with mixed source matrix, in which higher plants played an important role in forming weak reducing-weak oxidizing fresh water environment, and they have the geochemical characteristics of mature crude oil. All of these suggest that they are comparable.

It can be concluded from above-mentioned data that the crude oil of the Yanchang Formation and the source rocks of Chang 7 are comparable, which indicate that the crude oil is mainly derived from the source rocks of Chang 7 in the study area.

As shown in Table 1, the samples of crude oil are collected at the depth of 1614.2–2218.7 m; the samples of source rocks are collected at the depth of 1381.4–1970.6 m. The data of group component shows that, for crude oil samples, the content of saturated hydrocarbons is 43.75–76.95%, with an average of 63.37%; the content of aromatic hydrocarbon is 4.21–11.99%, with an average of 8.37%; the content of asphaltene + nonhydrocarbon is 15.40–44.26%, with an average of 28.26%; the value of saturated hydrocarbon/aromatic hydrocarbon is 3.65–17.27%, with an average of 8.47%.

Chang 7 source rock: the content of saturated hydrocarbons is 56.82–65.53%, with an average of 60.29%; the content of aromatic hydrocarbon is 5.37–11.92%, with an average of 8.52%; the content of asphaltene + nonhydrocarbon is 24.76–37.81%, with an average of 31.19%; the value of saturated hydrocarbon/aromatic hydrocarbon is 5.31–10.59%, with an average of 7.41%.

Other source rock: the content of saturated hydrocarbons is 15.88–53.67%, with an average of 35.62%; the content of aromatic hydrocarbon is 15.19–30.27%, with an average of 23.36%; the content of asphaltene + nonhydrocarbon is 31.13–63.54%, with an average of 41.01%; the value of saturated hydrocarbon/aromatic hydrocarbon is 0.77–3.53%, with an average of 1.68%.

The group components of the extract of Chang 7 source rock are significantly different from that of other source rocks but are similar to that of crude oil, so the samples of crude oil probably source from Chang 7 source rocks.

Based on the carbon isotopes of the monomeric compounds of the Mesozoic crude oil in the Ordos Basin and the results of oil source correlation, Zhang et al. [36, 37] considered that the crude oil of Yanchang Formation in the Ordos Basin was sourced from Yanchang Formation lacustrine source rocks. The results of this paper are consistent with that obtained from isotope study.

## 4. Conclusions

In this paper, saturated hydrocarbon fractions were extracted from crude oil and source rocks using organic geochemical methods. And the characteristics of the biomarker compounds in saturated hydrocarbon fractions were analyzed. These parameters have strong stability and good correlation. By comparing the characteristics of the biomarker compounds of crude oil and source rock samples, the depositional environment, sources, and maturity of the parent materials of crude oil were clarified and the main source rock beds were determined.

1. The parameters of biomarker compounds indicate that the crude oil of Yanchang Formation in Southwestern Yishan Slope is sourced from similar parent materials that are mixed type, and terrestrial higher plants play an important role. The crude oil is mature oil which is formed in weak reducing-weak oxidizing fresh or brackish water lake environment.
2. Based on the study of the geochemical characteristics of the crude oil of Yanchang Formation, the source rocks of Chang 7, and other source rocks in Southwestern of Yishan Slope, it is discovered that the geochemical characteristics of the crude oil of Yanchang Formation are similar, indicating the same source of the crude oil. According to the analysis of oil source correlation, the crude oil of Yanchang Formation derives from the source rocks of Chang 7 subformation.

## Figures and Tables

**Table 1:** Data of group component of crude oil and source rock samples in Southwestern Yishan Slope.

	Sample number	Reservoir	Deep (m)	Asp (%)	Sat (%)	Aro (%)	non (%)	Sat/Aro	Asp + non (%)
Crude oil	S1	Chang 1	1614.2	1.39	65.18	8.95	24.47	7.28	25.86
	S2	Chang 2	1671.7	1.32	56.51	10.42	31.76	5.42	33.08
	S3	Chang 2	1652.0	2.04	59.49	7.43	31.04	8.01	33.08
	S4	Chang 2	1643.0	1.40	58.57	10.17	29.86	5.76	31.26
	S5	Chang 3	1673.6	2.12	60.52	10.44	26.93	5.80	29.04
	S6	Chang 3	1686.0	1.16	62.19	9.56	27.09	6.51	28.25
	S7	Chang 4 + 5	1732.6	0.38	67.41	6.01	26.20	11.21	26.57
	S8	Chang 4 + 5	1712.0	1.33	56.19	10.05	32.44	5.59	33.77
	S9	Chang 6	1753.7	2.56	64.48	9.93	23.02	6.49	25.59
	S10	Chang 6	1796.1	6.26	53.04	8.90	31.81	5.96	38.07
	S11	Chang 7	1843.3	1.90	65.44	6.56	26.10	9.97	28.00
	S12	Chang 7	1827.0	0.42	76.95	5.98	16.66	12.87	17.08
	S13	Chang 8	1948.4	3.01	43.75	11.99	41.25	3.65	44.26
	S14	Chang 8	1879.1	1.58	63.64	10.94	23.84	5.81	25.42
	S15	Chang 8	1919.4	2.10	64.42	7.55	25.92	8.53	28.03
	S16	Chang 8	1955.7	0.82	76.95	7.69	14.54	10.01	15.36
	S17	Chang 9	2078.5	0.71	67.97	5.58	25.74	12.19	26.45
	S18	Chang 9	1974.2	0.63	72.69	4.21	22.46	17.27	23.10
	S19	Chang 10	2218.7	3.94	62.02	10.39	23.64	5.97	27.59
	S20	Chang 10	2124.0	0.74	69.94	4.61	24.70	15.16	25.44

Source rock	R1	Yan 6	1381.4	42.02	15.88	20.58	21.52	0.77	63.54
	R2	Yan 7	1419.1	20.55	26.52	26.62	26.31	1.00	46.87
	R3	Yan 9	1423.5	4.80	53.67	15.19	26.33	3.53	31.13
	R4	Chang 4 + 5	1689.1	21.92	28.59	30.16	19.33	0.95	41.25
	R5	Chang 6	1730.0	16.50	48.32	20.08	15.10	2.41	31.60
	R6	Chang 8	1945.2	22.79	40.00	20.63	16.58	1.94	39.37
	R7	Chang 9	1970.6	6.97	36.39	30.27	26.37	1.20	33.34
	R8	Chang 7	1723.4	0.58	59.40	7.86	32.16	7.56	32.74
	R9	Chang 7	1746.8	2.12	62.21	8.98	26.69	6.93	28.81
	R10	Chang 7	1813.3	1.87	57.65	9.42	31.06	6.12	32.93
	R11	Chang 7	1837.6	3.51	60.43	9.41	26.65	6.42	30.16
	R12	Chang 7	1823.7	2.63	57.98	6.15	33.24	9.43	35.87
	R13	Chang 7	1786.5	0.94	63.31	11.92	23.83	5.31	24.77
	R14	Chang 7	1803.3	4.14	65.53	9.59	20.74	6.83	24.88
	R15	Chang 7	1867.2	2.05	58.32	9.48	30.15	6.15	32.20
	R16	Chang 7	1783.7	1.93	61.27	6.99	29.81	8.76	31.74
	R17	Chang 7	1795.6	0.73	56.82	5.37	37.08	10.59	37.81

Asp: asphaltene; Sat: saturated hydrocarbon; Aro: aromatic hydrocarbon; non: nonhydrocarbon; Sat/Aro: saturated hydrocarbon/aromatic hydrocarbon; Asp + non: asphaltene + nonhydrocarbon.

**Table 2:** Comparative data of n-alkane and isoprenoid alkanes of samples in Southwestern Yishan Slope.

	Sample number	Reservoir	C_range_	OEP	∑C_21−_/∑C_22+_	Pr/Ph	Pr/*n*C_17_	Ph/*n*C_18_
Crude oil	S1	Chang 1	12~38	1.09	1.71	1.47	0.97	0.65
	S2	Chang 2	11~37	1.04	1.33	1.01	0.62	0.63
	S3	Chang 2	11~37	1.01	0.84	1.34	0.79	0.51
	S4	Chang 2	11~37	1.05	1.22	1.39	0.56	0.38
	S5	Chang 3	12~36	1.05	0.98	0.91	0.53	0.49
	S6	Chang 3	12~36	1.08	1.25	0.99	0.37	0.36
	S7	Chang 4 + 5	9~36	1.05	2.47	1.31	0.85	0.52
	S8	Chang 4 + 5	12~38	1.04	1.06	1.20	0.22	0.19
	S9	Chang 6	12~36	1.01	2.39	1.14	0.41	0.36
	S10	Chang 6	11~37	1.06	1.01	0.96	0.78	0.75
	S11	Chang 7	11~37	1.05	1.03	0.97	1.06	0.99
	S12	Chang 7	11~37	1.05	1.35	0.98	0.73	0.72
	S13	Chang 8	11~38	1.05	0.93	1.02	0.66	0.65
	S14	Chang 8	11~34	1.03	1.49	1.07	0.78	0.71
	S15	Chang 8	11~38	1.01	0.77	1.17	0.61	0.51
	S16	Chang 8	11~37	1.03	0.78	1.17	0.97	0.81
	S17	Chang 9	12~38	1.02	0.99	1.12	0.43	0.32
	S18	Chang 9	12~38	1.01	1.10	1.01	0.37	0.31
	S19	Chang 10	12~38	1.08	1.20	1.27	0.25	0.18
	S20	Chang 10	12~38	1.01	0.97	1.38	0.24	0.16

Source rock	R1	Yan 6	12~36	1.07	1.68	2.14	0.46	0.22
	R2	Yan 7	12~36	1.00	4.45	1.89	0.55	0.16
	R3	Yan 9	12~36	1.20	1.87	2.90	0.76	0.25
	R4	Chang 4 + 5	12~36	1.07	1.05	2.05	0.54	0.12
	R5	Chang 6	12~36	1.05	0.96	2.11	0.87	0.25
	R6	Chang 8	12~38	1.02	1.21	2.35	0.55	0.19
	R7	Chang 9	12~38	1.02	0.98	2.70	0.81	0.25
	R8	Chang 7	12~37	1.02	1.44	0.91	0.86	0.92
	R9	Chang 7	11~37	1.03	0.97	1.23	0.76	0.55
	R10	Chang 7	12~35	1.11	2.12	1.30	0.49	0.39
	R11	Chang 7	12~33	1.00	2.13	1.44	0.39	0.28
	R12	Chang 7	12~37	0.99	1.11	1.46	0.55	0.36
	R13	Chang 7	13~35	1.02	1.08	1.47	0.68	0.51
	R14	Chang 7	14~35	1.01	0.58	1.03	0.75	0.73
	R15	Chang 7	12~36	1.03	1.29	1.10	0.55	0.42
	R16	Chang 7	12~38	0.99	1.46	1.07	0.20	0.18
	R17	Chang 7	12~38	1.02	0.98	1.27	0.40	0.27

**Table 3:** Comparative data of steranes in crude oils and source rock samples in Southwestern Yishan Slope.

	Sample number	Reservoir	Regular Sterane abundance (%)			A	B	C	D	E	F
			C_27_	C_28_	C_29_						
Crude oil	S1	Chang 1	23.1	29.4	47.5	0.19	0.38	0.13	0.51	0.55	0.54
	S2	Chang 2	24.2	28.1	47.7	0.25	0.18	0.16	0.51	0.59	0.51
	S3	Chang 2	26.4	29.7	43.9	0.25	0.22	0.14	0.52	0.55	0.52
	S4	Chang 2	27.1	28.6	44.3	0.26	0.19	0.14	0.49	0.53	0.45
	S5	Chang 3	26.1	28.2	45.7	0.26	0.11	0.12	0.53	0.59	0.69
	S6	Chang 3	25.9	28.9	45.2	0.24	0.07	0.11	0.52	0.61	0.70
	S7	Chang 4 + 5	31.1	31.2	37.7	0.74	0.29	0.11	0.52	0.53	0.69
	S8	Chang 4 + 5	28.1	28.0	43.9	0.25	0.75	0.14	0.54	0.51	0.68
	S9	Chang 6	25.3	36.0	38.7	0.29	0.19	0.14	0.54	0.59	0.67
	S10	Chang 6	23.6	28.7	47.7	0.25	0.23	0.15	0.52	0.59	0.55
	S11	Chang 7	22.1	27.4	50.5	0.16	0.18	0.13	0.50	0.56	0.52
	S12	Chang 7	25.9	29.3	44.8	0.26	0.24	0.14	0.55	0.58	0.56
	S13	Chang 8	23.0	30.1	46.9	0.28	0.16	0.20	0.51	0.62	0.54
	S14	Chang 8	26.5	29.7	43.8	0.34	0.21	0.16	0.55	0.61	0.55
	S15	Chang 8	23.7	29.9	46.4	0.19	0.14	0.21	0.53	0.58	0.52
	S16	Chang 8	24.2	29.9	45.9	0.19	0.17	0.20	0.53	0.50	0.47
	S17	Chang 9	28.0	23.0	49.0	0.64	0.1	0.11	0.55	0.55	0.73
	S18	Chang 9	27.0	27.0	46.0	0.8	0.07	0.08	0.53	0.63	0.62
	S19	Chang 10	30.0	30.0	40.0	0.64	0.21	0.11	0.53	0.54	0.69
	S20	Chang 10	28.0	28.0	44.0	0.28	0.18	0.14	0.57	0.53	0.77

Source rock	R1	Yan 6	18.0	17.0	65.0	0.12	1.82	0.14	0.45	0.43	0.60
	R2	Yan 7	15.0	16.0	69.0	0.05	0.20	0.13	0.45	0.43	0.59
	R3	Yan 9	16.0	17.0	67.0	0.09	0.15	0.16	0.44	0.35	0.57
	R4	Chang 4 + 5	24.0	22.0	54.0	0.13	0.56	0.15	0.55	0.52	0.58
	R5	Chang 6	21.0	19.0	60.0	0.13	0.61	0.12	0.53	0.51	0.56
	R6	Chang 8	23.0	23.0	54.0	0.63	0.06	0.11	0.51	0.51	0.72
	R7	Chang 9	23.0	19.0	58.0	0.68	0.24	0.14	0.47	0.47	0.73
	R8	Chang 7	30.8	28.7	40.5	0.37	0.37	0.16	0.56	0.57	0.61
	R9	Chang 7	27.7	20.8	51.5	0.12	0.34	0.16	0.55	0.54	0.59
	R10	Chang 7	29.4	25.1	45.5	0.17	0.20	0.16	0.53	0.53	0.46
	R11	Chang 7	30.7	23.2	46.1	0.22	0.22	0.16	0.54	0.54	0.58
	R12	Chang 7	26.9	24.9	48.2	0.18	0.26	0.16	0.55	0.56	0.60
	R13	Chang 7	30.0	24.1	45.9	0.28	0.48	0.21	0.54	0.52	0.58
	R14	Chang 7	29.6	20.6	49.8	0.29	0.52	0.15	0.48	0.54	0.61
	R15	Chang 7	29.0	21.0	50.0	0.53	0.39	0.10	0.47	0.61	0.56
	R16	Chang 7	27.0	31.0	42.0	0.31	0.02	0.15	0.48	0.51	0.71
	R17	Chang 7	32.0	19.0	49.0	0.89	0.17	0.06	0.51	0.45	0.78

A-∑regular Steranes/∑Hopanes; B-∑diasteranes/∑regular Steranes; C-∑4-Methyl Steranes/∑regular Steranes; D-C_29_ Steranes 20S/(20S + 20R); E-C_29_ Steranes *ββ*/(*αα* + *ββ*); F-C_29_ diasteranes 20S/(20S + 20R).

**Table 4:** Comparative data of Terpanes in crude oil and source rock samples in Southwestern Yishan Slope.

	Sample number	Reservoir	A	B	C	D	E	F	G
Crude oil	S1	Chang 1	0.72	0.51	0.46	21.5	2.14	0.04	0.57
	S2	Chang 2	0.76	0.50	0.51	22.5	0.55	0.05	0.56
	S3	Chang 2	0.80	0.52	0.49	25.9	0.65	0.04	0.56
	S4	Chang 2	0.76	0.52	0.48	26.5	0.70	0.05	0.58
	S5	Chang 3	0.70	0.50	0.38	26.4	0.89	0.05	0.57
	S6	Chang 3	0.71	0.47	0.44	26.3	0.78	0.05	0.58
	S7	Chang 4 + 5	0.67	0.49	0.44	24.8	1.03	0.04	0.57
	S8	Chang 4 + 5	0.68	0.42	0.35	23.0	9.79	0.04	0.52
	S9	Chang 6	0.69	0.42	0.45	23.2	0.56	0.06	0.54
	S10	Chang 6	0.72	0.53	0.48	22.6	0.52	0.05	0.57
	S11	Chang 7	0.68	0.40	0.48	20.3	0.32	0.03	0.57
	S12	Chang 7	0.71	0.48	0.50	24.2	0.59	0.05	0.55
	S13	Chang 8	0.64	0.30	0.38	23.4	0.76	0.06	0.59
	S14	Chang 8	1.05	0.43	0.46	21.3	1.00	0.05	0.55
	S15	Chang 8	0.44	0.42	0.45	23.8	0.93	0.04	0.58
	S16	Chang 8	0.58	0.38	0.45	21.1	0.89	0.06	0.56
	S17	Chang 9	0.69	0.44	0.26	28.0	5.86	0.04	0.42
	S18	Chang 9	1.03	0.47	0.23	24.0	1.23	0.02	0.54
	S19	Chang 10	0.98	0.45	0.25	19.0	6.46	0.04	0.45
	S20	Chang 10	0.78	0.45	0.35	22.0	4.26	0.05	0.55

Source rock	R1	Yan 6	2.57	0.96	0.81	29.0	0.05	0.03	0.59
	R2	Yan 7	3.14	0.56	0.76	40.0	0.03	0.02	0.58
	R3	Yan 9	3.75	0.72	0.81	28.0	0.04	0.03	0.57
	R4	Chang 4 + 5	1.71	0.61	0.64	29.0	3.61	0.06	0.59
	R5	Chang 6	3.03	0.68	0.81	30.0	0.70	0.05	0.60
	R6	Chang 8	1.05	0.58	0.48	20.0	0.96	0.10	0.60
	R7	Chang 9	1.96	0.46	0.23	26.0	4.45	0.10	0.47
	R8	Chang 7	0.88	0.38	0.47	32.1	3.92	0.13	0.54
	R9	Chang 7	1.14	0.53	0.65	27.4	0.94	0.03	0.57
	R10	Chang 7	0.90	0.40	0.58	25.7	1.29	0.04	0.56
	R11	Chang 7	1.51	0.43	0.70	18.4	1.30	0.03	0.57
	R12	Chang 7	0.78	0.45	0.49	27.2	0.98	0.04	0.56
	R13	Chang 7	1.56	0.33	0.40	31.6	3.89	0.08	0.56
	R14	Chang 7	1.38	0.50	0.45	35.7	2.63	0.08	0.58
	R15	Chang 7	1.45	0.52	0.30	31.0	2.51	0.12	0.61
	R16	Chang 7	1.00	0.52	0.52	20.0	3.74	0.07	0.58
	R17	Chang 7	0.95	0.53	0.47	26.0	1.71	0.08	0.57

A-(C_19_ tricyclic Terpanes + C_20_ tricyclic Terpanes)/C_23_ tricyclic Terpanes; B-C_25_ tricyclic Terpanes/C_26_ tricyclic Terpanes; C-C_24_ tetracyclic Terpanes/(C_24_ tetracyclic Terpanes + C_26_ tricyclic Terpanes); D-*∑* > C_30_ Hopanes abundance (%); E-Ts/Tm; F-Gammacerane/*αβ*-C_30_ Hopanes; G-C_31_ Hopanes 22S/(22S + 22R).
